# Primary Dysmenorrhea in Relation to Oxidative Stress and Antioxidant Status: A Systematic Review of Case-Control Studies

**DOI:** 10.3390/antiox9100994

**Published:** 2020-10-15

**Authors:** Maria Karolina Szmidt, Dominika Granda, Ewa Sicinska, Joanna Kaluza

**Affiliations:** Institute of Human Nutrition Sciences, Warsaw University of Life Sciences–SGGW, 159C Nowoursynowska Str., 02-776 Warsaw, Poland; dominika_granda@sggw.edu.pl (D.G.); ewa_sicinska@sggw.edu.pl (E.S.); joanna_kaluza@sggw.edu.pl (J.K.)

**Keywords:** antioxidant status, inflammation, oxidative stress, primary dysmenorrhea, women

## Abstract

Primary dysmenorrhea is defined as painful menstrual cramps of uterine origin in the absence of pelvic pathology and is the most common gynecological disorder among women of reproductive age. The aim of this study was to systematically review case-control studies that have investigated the oxidative stress, antioxidant status, and inflammation markers among women with primary dysmenorrhea and controls. The study protocol was registered with PROSPERO (no. CRD42020183104). By searching PubMed and Scopus databases as well as reference lists, six case-control studies with fifteen eligible markers (seven oxidative stress, seven antioxidant status, one inflammation) were included in this review. The quality of the included studies was assessed as medium or high. The systematic review included 175 women with primary dysmenorrhea and 161 controls. The results indicate an elevated level of oxidative stress, especially of lipid peroxidation among dysmenorrheal women. For the antioxidant status, limited evidence was found for a lower status among primary dysmenorrhea women, and only one study examined one inflammation marker (hs-CRP), which makes it impossible for such a conclusion. To establish whether oxidative stress, antioxidant status or inflammation participate in the pathophysiology of primary dysmenorrhea, high-quality studies with larger study groups and clear case definitions are needed.

## 1. Introduction

Dysmenorrhea, defined as painful menstrual cramps of uterine origin, is one of the most common causes of pelvic pain and menstrual disorder [1]. According to ICD-10 [2], there are two types of dysmenorrhea—primary dysmenorrhea, which is characterized by a lack of organic disease, and secondary dysmenorrhea, associated with an identifiable disease, e.g., endometriosis, fibroids, adenomyosis [3]. Primary dysmenorrhea is defined as painful, spasmodic cramping in the lower abdomen, just before and/or during menstruation in the absence of pelvic pathology [4]. Usually, the pain lasts for 8–72 h just before or at the start of menstruation, has a clear and predictable temporal pattern, and onset occurs in adolescence, within 6–12 months from menarche [4,5,6,7]. The most severe pain is observed during the first or second day of menstruation and is accompanied by symptoms that include diarrhea, nausea, vomiting, headaches, and others [3,6,8]. Globally, primary dysmenorrhea affects 50–90% of menstruating women, and it is also the most common gynecological disorder reported by them, irrespective of nationality or age [6,9]. Additionally, primary dysmenorrhea has been classified as a chronic pelvic pain syndrome [10], and it is observed as a frequent comorbidity with other painful conditions [11], e.g., irritable bowel syndrome, fibromyalgia, and migraine [12,13,14].

However, it is considered that the prevalence of primary dysmenorrhea may be highly underestimated, due to delay in accessing medical care, as many women consider pain as a normal part of the menstrual cycle [7]. Moreover, the lack of standard methods for assessing the severity of dysmenorrhea and also its inadequate treatment by some physicians worsen these statistics [6,9,15]. Quick diagnosis and treatment are crucial because primary dysmenorrhea has a negative impact on the quality of life during menstruation, mood, quality of sleep, and is frequently the reason for absence from school or work [4,8,9,15].

The pathogenesis of primary dysmenorrhea is not fully understood, but the most widely accepted explanation is based on the overproduction of uterine prostaglandins [4,8,15]. Progesterone withdrawal at the late luteal phase leads to the secretion of arachidonic acid and release of prostaglandins (especially PGF2a and PGE2) and leukotrienes during endometrial sloughing, which may cause myometrial hypercontractility. Moreover, vasoconstriction and myometrial contractions result in the ischemia and hypoxia of the uterine muscle, which causes pain and systemic symptoms [6,16,17]. During the luteal phase, increased levels of prostaglandins (compared to the follicular phase) are observed in all women; however, dysmenorrheal women have higher levels of these hormones than controls, especially during the first two days of menses [1,4,8]. Other potential mechanisms that can explain primary dysmenorrhea’s etiology involve vasopressin levels, cytokine gene expression profiles, prolactin levels in the luteal phase, nocturnal body temperatures, and morning estrogen concentrations [6]. Considering vasopressin levels in primary dysmenorrhea etiology remains controversial, as only a limited number of studies showed elevated levels of this hormone during menstruation among women with primary dysmenorrhea [18,19]. Hypothetically, higher vasopressin levels may cause dysrhythmia uterine contraction and may result in further uterine hypoxia, ischemia, and contribute to pain intensity. It is also proposed that dysmenorrheal women may be characterized by a different inflammatory response. Ma et al. showed that, compared with controls, women with primary dysmenorrhea demonstrated upregulation of genes related to pro-inflammatory cytokines and the simultaneous downregulation of genes related to anti-inflammatory response [20]. Moreover, in some studies, women with primary dysmenorrhea had elevated prolactin levels in the luteal phase, which may play an etiological role in dysmenorrhea [6,21]. In addition, a very limited number of studies considered higher nocturnal body temperature and increased morning estrogen concentrations during the menstrual cycle among women with primary dysmenorrhea compared with healthy ones as possible etiology mechanisms [22].

Oxidative stress, which is linked to the pathogenesis of more than 100 diseases [23,24], may play a substantial role in endothelial dysfunction including uterine endothelium [25,26,27,28] and the severity of dysmenorrhea [29,30]. Therefore, oxidative stress and antioxidant status constitute another potential mechanism of primary dysmenorrhea. The counteracting of an imbalance between reactive oxygen species (ROS) production and the ability to detoxify these products by the human body is based on endogenous or exogenous antioxidants [24]. Endogenous antioxidants are produced by an organism in enzymatic, as well as nonenzymatic, forms, while exogenous antioxidants are delivered with diet and nutritional supplements [24]. Understanding the link between oxidative stress and antioxidant status in relation to primary dysmenorrhea seems to be crucial in its prevention, diagnosis, and treatment. Potentially, if oxidative stress plays a role among other factors in the severity of menstrual pain, antioxidant therapy could be considered in further therapeutic interventions.

To the best of our knowledge, the association between oxidative stress, antioxidant status, inflammation markers, and primary dysmenorrhea has not been previously systematically assessed. We, thus, aimed to conduct a comprehensive systematic review to summarize available data on the association between oxidative stress, antioxidant status, inflammation markers, and primary dysmenorrhea.

## 2. Materials and Methods

The protocol for this review has been submitted for registration in the International Prospective Register of Systematic Reviews (identification number CRD42020183104).

### 2.1. Search Strategy

A systematic review of the literature was conducted following the Preferred Reporting Items for Systematic Review and Meta-Analysis (PRISMA) guidelines [31]. PubMed and Scopus databases have been searched from inception to 8 May 2020 without language restrictions. To identify potential studies, a combination of terms related to ‘primary dysmenorrhea’, ‘oxidative stress’, ‘antioxidant status’, and ‘inflammation’ was used. Details on the search strategy are shown in Table S1. In addition, the reference lists of the identified publications were searched manually for further indication of potentially eligible studies.

### 2.2. Inclusion and Exclusion Criteria

The inclusion criteria consisted of: (1) A case-control study design; (2) studies where women had regular menstrual cycles; (3) studies where women with primary dysmenorrhea were compared to controls in relation to circulating markers of oxidative stress and/or antioxidant status and/or inflammation; (4) studies with information on how primary dysmenorrhea was diagnosed; and (5) articles written in English.

The exclusion criteria consisted of: (1) A different study design than case-control; (2) studies where women were diagnosed with gynecological or/and oxidative stress diseases (endometriosis, amenorrhea, irritable bowel syndrome, diabetes, etc.), sexually transmitted diseases, or chronic pelvic pain caused by reasons other than primary dysmenorrhea; and (3) studies with a lack of information on how primary dysmenorrhea was diagnosed.

### 2.3. Study Selection and Data Extraction

Titles and abstracts of the identified studies were screened by two independent reviewers (M.K.S. and D.G.) for eligibility. After that, a detailed full-text evaluation was performed using the inclusion and exclusion criteria. In the event of noncompliance, the eligibility of an article was discussed, and the decision was made by a next co-author (J.K.). One author (M.K.S.) abstracted data from eligible studies using a predesigned data collection form. The other author (D.G.) independently checked the correctness of these data based on original articles. Disagreements were discussed and resolved with the involvement of other co-authors (J.K. and E.S.). In the event of a lack of some necessary information in the potentially eligible studies, the authors were contacted in order to obtain such information.

From the eligible case-control studies, the following information was extracted: the first author’s last name, publication year, the country where the study was performed, number of cases and control group size, used definition of primary dysmenorrhea, participants’ characteristics, mean and standard deviation (SD) of age, and biomarkers of interest, type of biologic sample, mean and SD or median (interquartile range) of the analyzed biomarkers, *p*-value between cases and control groups, and information about adjustment for potential confounders (defined as “yes” or “no”).

### 2.4. Quality Assessment

The quality of the studies was assessed using the Newcastle–Ottawa Quality Assessment Scale (NOS) for case-control studies [32]. The NOS is recommended by the Cochrane Collaboration for use in nonrandomized studies [33]. The NOS rates studies according to three pre-defined criteria: selection, comparability, and exposure, where nine points reflect the highest quality of case-control studies.

## 3. Results

### 3.1. Description of Included Studies

**Figure 1:**
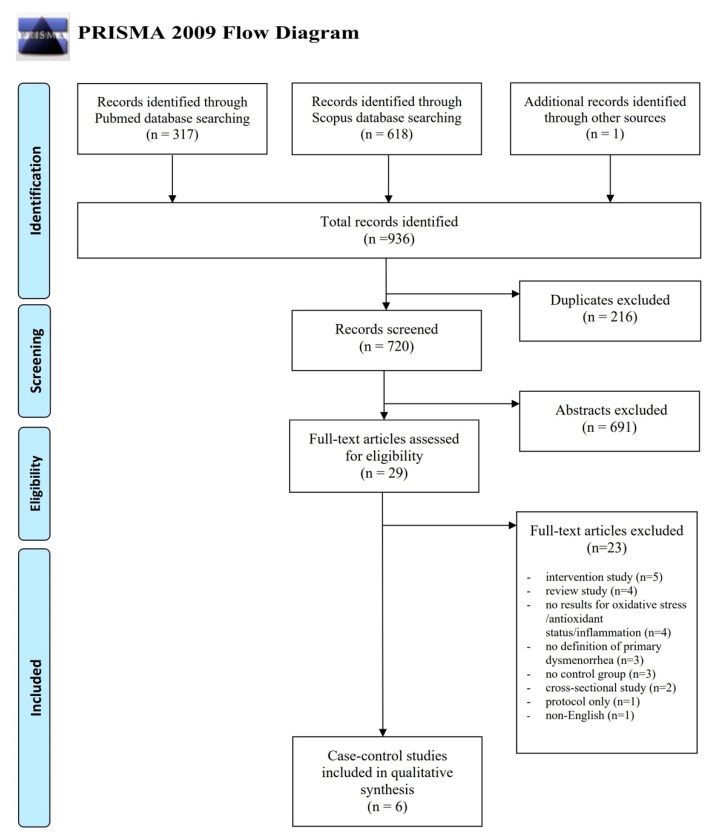
Literature review flow diagram of the selection process according to the PRISMA Statement [31].

We identified 935 studies through database searches and one additional publication via manual searching of the reference lists, of which 216 were duplicates (Figure 1). From 720 publications, 691 were excluded based on screening titles and abstracts, and 29 publications were classified for full-text article assessment. A total of six case-control studies met the inclusion criteria and was eligible for our review.

The eligible studies were published between 2008 and 2019. The main characteristics of the included studies are reported in Table 1. Five studies were conducted among Turkish women [30,34,35,36,37], and one study among Nigerian women [38]. The sample size ranged from 12 [37] to 90 [38] women, with a total of 175 primary dysmenorrhea cases. The range of mean age was from 20 [36] to 27 [35] years. The diagnosis of primary dysmenorrhea was based on a variety of methods and criteria, which included gynecologic examination, visual analog scale (VAS) of pain, and self-reported indication.

### 3.2. Study Quality

The methodological quality assessment of the studies indicated that two case-control studies were of high quality [35,38], and the remaining four studies were of medium quality (Table 2) [30,34,36,37]. None of the six studies has been adjusted for important potential confounding factors, such as age, physical activity, smoking status, body mass index. Details of the quality assessment of the studies are shown in Table S2.

### 3.3. Oxidative Stress and Inflammation

#### 3.3.1. Lipid Peroxidation

Four case-control studies examined the malondialdehyde (MDA) concentration in the blood of women with primary dysmenorrhea and controls (Table 3) [30,35,36,38]. In three studies, the concentration of MDA was measured in plasma [30,36,38], and in one, the concentration was measured in serum [35]. Considering both serum and plasma concentrations, women with primary dysmenorrhea had a statistically significant higher level of MDA than controls. In two studies (one of high [38] and one of medium [30] quality), which provided results of MDA concentration in nmol/mL of plasma, mean results were in the range of 0.75–1.32 nmol/mL for women with primary dysmenorrhea and 0.45–0.91 nmol/mL for control groups.

In a study of medium quality and small sample size, the lipid peroxidation (LP) level was examined [37]. The study showed that, compared to controls, women with primary dysmenorrhea had a significantly higher LP level (6.97 ± 0.51 vs. 5.63 ± 0.73 μmol/g protein).

#### 3.3.2. Protein and DNA Damage

Two studies examined protein damage markers in relation to primary dysmenorrhea—Table 3 [30,38]. In the high-quality study, compared to controls, women with primary dysmenorrhea had a statistically significant higher 3-nitrotyrosine (3-NT) level (45.9 ± 37.1 vs. 21.3 ± 13.9 ng/mL), but not protein carbonyl groups [38]. In the other study of medium quality, a difference in the 3-NT concentration between women with dysmenorrhea and those in the control group was not observed [30].

Only one study analyzed the concentration of 2′-deoxy-8-hydroxy-guanosine (8-OhdG), the major product of DNA oxidation, in relation to primary dysmenorrhea [30]. The serum concentration of 8-OhdG was lower in women with dysmenorrhea versus the control group; the obtained difference was on the border of statistical significance.

#### 3.3.3. Other Oxidative Stress and Inflammation Markers

Two studies (one of high [35] and second of medium [36] quality) assessed nitric oxide (NO) levels in the blood of women with primary dysmenorrhea and in healthy women (Table 3). In both studies, women with primary dysmenorrhea had a significantly higher level of NO compared to controls.

Only in one study of medium quality was the concentration of asymmetric dimethylarginine (ADMA) in serum compared between women with primary dysmenorrhea and controls, with a statistically significant higher concentration of ADMA in the dysmenorrhea group (median 0.31 µmol/L) versus the control group (mean 0.27 μmol/L) [34].

Despite the broad search strategy, we identified only one study [34], which examined one inflammation marker strongly associated with oxidative stress—high-sensitivity C-reactive protein (hs-CRP). However, a statistically significant difference in the hs-CRP concentration between women with primary dysmenorrhea and control ones were not found [34].

### 3.4. Antioxidant Status

The total antioxidant status (TAS) was examined in one small sample size study of medium quality in neutrophil (Table 4) [37]. Women with primary dysmenorrhea had statistically significant lower levels of TAS compared to controls (3.77 ± 0.55 vs. 4.98 ± 1.19 μmol H_2_O_2_ equivalent/g protein).

#### 3.4.1. Enzymatic Markers

The activity of three different enzymatic markers among women with primary dysmenorrhea and control ones were examined in three studies [30,35,37]. The medium-quality study examined the superoxide dismutase (SOD) activity in women with primary dysmenorrhea and controls, and no significant differences between the groups were found [30].

Considering other antioxidant enzymes, the concentration of heme oxygenase 1 (HO1) was examined in one study of high quality [35], and the activity of glutathione peroxidase (GSH-Px) was examined in another study of medium quality [37]. HO1 concentration was significantly higher in women with primary dysmenorrhea compared to the control group (5.36 ± 1.57 vs. 3.94 ± 0.97 ng/mL) [35], while GSH-Px activity was significantly lower in women with primary dysmenorrhea than in controls (4.07 ± 0.62 vs. 4.74 ± 0.99 IU/g protein) [37].

#### 3.4.2. Non-Enzymatic Markers

In one study of medium quality, glutathione levels (GSH) were examined, and no statistically significant differences were found between groups (Table 4) [37]. In the other medium-quality study, adrenomedullin (AM) levels were assessed [36]. Women with primary dysmenorrhea had statistically significant higher plasma levels of AM compared to controls.

Only one high-quality study examined the vitamin E level in the blood of women with primary dysmenorrhea and healthy controls [38]. Women with primary dysmenorrhea had a statistically significant lower plasma level of vitamin E compared to the control group (7.51 ± 1.95 vs. 8.98 ± 1.95 µmol/L) [38].

## 4. Discussion

Our systematic review of six case-control studies indicates that the levels of the most oxidative stress markers were significantly higher in women with primary dysmenorrhea compared to controls. For the antioxidant status, limited evidence was found on the lower status in primary dysmenorrhea women and the number of studies that analyzed antioxidant markers is insufficient to make clear conclusions. Moreover, only one case-control study examined one inflammation marker (hs-CRP), which makes it impossible to draw conclusions in this area.

The results of our systematic review showed that the findings of several studies are consistent and indicate an elevated level of different oxidative stress markers among women with primary dysmenorrhea compared to controls. MDA levels (one of the end products of lipid peroxidation) [39] were higher among women with primary dysmenorrhea compared to controls in all studies included in the review [30,35,36,38]. These results are in line with the other case-control study excluded from this systematic review due to no case definition and no description of inclusion and/or exclusion criteria [40]. In this study, the MDA concentration in serum was also higher in women with primary dysmenorrhea than in the control group (4.31 ± 0.48 vs. 1.95 ± 0.23 nmol/mL) [40]. Moreover, in another study included in this review, LP levels were also significantly higher among women with primary dysmenorrhea compared to controls [37]. All of these results indicate an elevated level of lipid peroxidation in patients experiencing primary dysmenorrhea.

Consistent results were also found for NO, one of the reactive nitrogen species produced by endothelial [41]. In two studies, higher levels of NO were found among primary dysmenorrhea women compared to controls [35,36]. These results are in line with a case-control study from Taiwan (serum NO levels 0.36 ± 0.02 in a dysmenorrhea group vs. 0.28 ± 0.02 ppm in controls), which was not eligible for this review due to no case definition and no description of inclusion and/or exclusion criteria [42]. However, these results contradict the other prospective controlled trial (not included in this review), where no significant differences in the levels of NO were found among women with primary dysmenorrhea and controls at baseline as well as after providing an eight-week yoga intervention [43]. Considering endothelial dysfunction, in the study conducted by Akdemir et al. (included in this systematic review), it was found that women with primary dysmenorrhea had higher levels of ADMA compared to controls [34]. Along with the results for the NO levels, this may indicate that endothelial dysfunction may play a role in this disorder.

Our systematic review also showed conflicting results for some oxidative stress markers. We have found inconsistent results for 3-NT levels, a marker of nitration damage to protein [44]. In one study, women with primary dysmenorrhea had significantly higher (more than twice) 3-NT levels compared to controls [38], but no such conclusions were made in the other study [30]. 3-NT levels were measured in both studies in plasma using enzyme-linked immunosorbent assay (ELISA) kit, but it was from different manufacturers. Additionally, the difference in obtained results may at least partially be due to differences in total and group sample sizes (cases vs. controls: n = 45 vs. n = 45 [38] and n = 33 vs. n = 25 [30]) and differences in mean age in primary dysmenorrhea groups (22.5 ± 3.3 [38] vs. 24.2 ± 3.1 [30] years) and control groups (21.8 ± 2.8 [38] vs. 25.0 ± 2.9 [30] years). Although a significant difference in 3-NT levels was found by Orimadegun et al. [38], no significant differences in PrCarb levels were observed, which may suggest the absence of excessive protein oxidation among women with primary dysmenorrhea. Turhan et al. also examined the 8-OhdG concentration (a marker of oxidative DNA damage) with no significant differences in its level between primary dysmenorrhea and the control group; however, women with primary dysmenorrhea had a tendency toward higher 8-OhdG levels [30]. It is possible that the observed tendency (*p*-value = 0.051) may be biologically important. Therefore, further research is required to establish whether protein and DNA oxidation takes place among dysmenorrheal women.

Only in one study was hs-CRP (inflammation marker) examined and there was no significant difference observed in its level between women with primary dysmenorrhea and controls [34]. Due to the well-known relation between oxidative stress and inflammation [45,46], it seems important to perform further studies covering the topic of inflammation among women with primary dysmenorrhea, using various inflammation markers.

In this systematic review of case-control studies, limited evidence was found on associations of the antioxidant body status with primary dysmenorrhea women. The limited number of studies and insufficient comprehensive analysis of antioxidant markers did not allow to make a conclusion on the relationship between a low antioxidant status and risk of primary dysmenorrhea. In one study, women with primary dysmenorrhea had significantly lower levels of the total antioxidant status marker and lower activity of GSH-Px compared to controls [37]. Additionally, serum activity of HO1, an enzyme with anti-oxidative and anti-inflammatory properties [35], and serum levels of AM, which has a protective role against oxidative stress [36], were observed significantly higher among women with primary dysmenorrhea compared to controls. However, no significant differences were observed for SOD [30] and GSH [37].

In one study, women with primary dysmenorrhea had significantly lower levels of vitamin E (α-tocopherol) than controls [38]. The results obtained by Orimadegun et al. [38] are in line with another case-control study not included in this systematic review, where levels of vitamin E were significantly lower in women with primary dysmenorrhea compared to controls (0.78 ± 0.07 vs. 1.34 ± 0.29 mg%) [40]. Furthermore, the results of a randomized, double-blind, placebo-controlled trial (not included in this review) indicate that the treatment of vitamin E relieves the primary dysmenorrhea pain [47]. After 4 months of vitamin E treatment (200 IU twice a day, two days before menstruation and the first three days of bleeding) women from intervention vs. placebo group had lower pain severity (0.5 vs. 6.0 in the 10-point scale, *p*-value < 0.001) and shorter pain duration (1.6 vs. 16.7 h, *p*-value < 0.001) [47].

None of the studies included in this systematic review examined vitamin C levels in relation to primary dysmenorrhea. However, in the case-control study (mentioned previously and not included in this review), levels of vitamin C in plasma were significantly lower among women with primary dysmenorrhea compared to controls (0.68 ± 0.08 vs. 1.14 ± 0.19 mg%, *p*-value = 0.001) [40].

The presented results indicate that women with primary dysmenorrhea may have a deficiency of antioxidant vitamins, which can potentially explain higher levels of oxidative stress markers among them. Lower consumption of foods with high antioxidant potential, especially fruits and vegetables, may be associated with a high prevalence of primary dysmenorrhea. In two cross-sectional studies [48,49], but not in another one [50], higher consumption of fruits and vegetables was observed among dysmenorrheic cases compared to controls. Tavallaee et al. found an association between the level of menstrual pain and the consumption of fruits and vegetables; women with high and very high consumption vs. those with never or low consumption had, respectively, 60% and 80% lower level of menstrual pain [48]. Therefore, it is plausible that the observed low antioxidant status among dysmenorrheal women may be at least partially explained by the low consumption of foods with high antioxidant potential. Moreover, lowered antioxidant status in dysmenorrheal women may be a result of reduced activity of cellular endogenous antioxidant enzymes (such as SOD, GSH-Px, etc.) as well as lowered level of endogenus non-enzymatic antioxidants (for example, metallothioneins, glutathione, ceruloplasmin, etc.). A lowered antioxidant status may also be an effect of reduced intake or absorption of exogenous antioxidants (from food or supplements), for example, due to the presence of undiagnosed diseases, genetic predisposition, and others [51].

None of the studies included in this systematic review examined or discussed the natural fluctuations in oxidative stress, antioxidant status, or inflammatory markers across the whole menstrual cycle among dysmenorrheal women. However, the variation in oxidative stress and antioxidant markers was examined in other studies [52,53]. Cornelli et al. showed the maximum lipid peroxidation levels measured by hydroperoxides in plasma near the estrogen peak at the end of the follicular phase and slowly returning to base levels at the end of the menstrual cycle in a group of 20 healthy women [52]. These results are also in line with a study from Poland, where the urinary levels of hydrogen peroxide and thiobarbituric acid were significantly higher across the luteal phase compared to the follicular phase [53]. Furthermore, Michos et al. showed the variation in the total antioxidant capacity through the menstrual cycle, suggesting elevated antioxidant protection during ovulation and the mid-luteal phase [54].

### 4.1. Potential Mechanism

**Figure 2:**
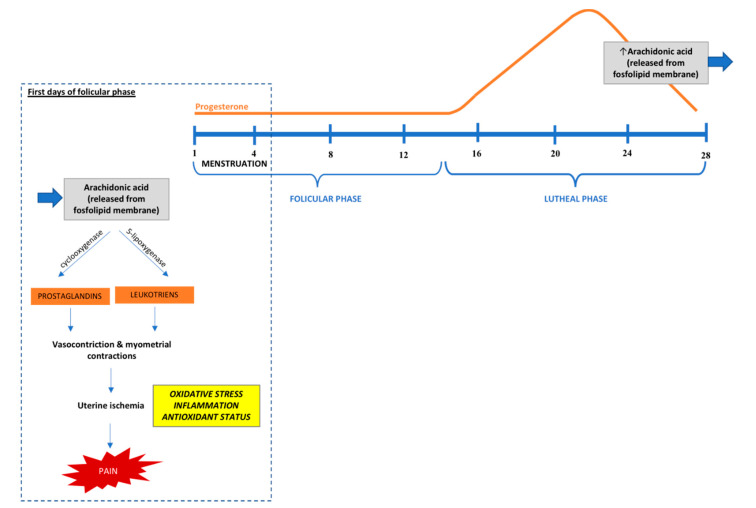
Literature review flow diagram of the selection process according to the PRISMA statement [17].

Although a great deal of research has been conducted on primary dysmenorrhea, its pathomechanism is still not fully understood. As early as 1986, menstruation has been described as an inflammatory event [55], and later studies showed that menstrual cycle disturbances might be related to the inflammatory mechanism [56]. Two years later, it has been suggested that inflammation and endothelial dysfunction may be characterized by lipid peroxidation [25]. Nowadays, one of the convincing mechanisms that stand behind elevated markers of inflammation is based on the role of progesterone in the menstrual cycle [8,16] (Figure 2). During the first half of secretory (luteal) phase in the menstrual cycle, the level of progesterone increases, which has anti-inflammatory and regulating (prostaglandins and leukocytes synthesis) effects on endometrial tissue [8,16]. In the second half of the luteal phase, the progesterone level begins to fall, which causes the secretion of arachidonic acid and its metabolites, such as prostaglandins and leukotrienes [6,12,16,57]. Prostaglandin and leukotrienes cause vasoconstriction and myometrial contraction, leading to uterine ischemia, which results in pain and also other complaints, such as vomiting or headache [6,17]. It was reported that in the case of ischemia oxidative stress occurs [39,40,41], which may play a significant role in the etiopathogenesis of primary dysmenorrhea [35]. Furthermore, it is currently well established that inflammation and oxidative stress are inseparably interrelated [45,58], and ROS that are produced as part of the inflammatory response are the primary source of oxidative damage, which can impair lipid, protein, and nucleic acid [59]. Thus, antioxidants may play an important role in primary dysmenorrhea by reducing inflammation. For example, by inhibiting the release of arachidonic acid and its conversation to prostaglandins, vitamin E may play a role in reducing pain [47,60,61]. Additionally, zinc, besides having antioxidant and anti-inflammatory properties, takes part in reducing prostaglandin metabolism and may decrease the level of pain [62,63].

Furthermore, the intensity of pain perception may be influenced by previous pain experiences, mood, stress, sleeping disorders, the occurrence of premenstrual syndrome, and some cultural and social influences [6,16]; whilst oxidative stress may also be modulated by lifestyle and environmental factors, such as diet, physical activity, alcohol consumption, smoking cigarettes, medical drug abuse, sleep deprivation, stress, or environmental pollution (pesticides, heavy metals, xenoestrogens, etc.) [64,65,66] Thus, similar factors may impact both pain and oxidative stress. Therefore, it cannot be excluded that the potential mechanism that stands behind primary dysmenorrhea and ROS balance is modulated by similar common factors.

### 4.2. Strengths and Limitations

The strengths of our systematic review consist of clearly defined inclusion criteria and strict compliance with procedures for performing a systematic review. The actual number of potentially relevant studies was higher than six, but after assessment for eligibility and not being able to complement data, we decided to exclude one case-control study in the final step of performing the systematic review search [40]. The paper was excluded due to no case definition and no description of inclusion and/or exclusion criteria [40]. That study reported a statistically significant association between primary dysmenorrhea and the following markers: MDA, SOD, GSH, vitamin E, and vitamin C [40], which may indicate higher oxidative stress and lower antioxidant status of dysmenorrheal women.

Furthermore, one of the strengths of our study is that the broad search strategy of databases was conducted by two independent researchers. Next, a quality assessment of the included studies was performed; the systematic review includes medium- or high-quality studies, and none of them was of low quality. However, none of the studies was adjusted for potential confounding factors, such as age or BMI, which may lead to false-positive results. We have also observed a substantial variation in some methodological aspects of the included studies. The majority of studies assessed primary dysmenorrhea based on the clinical history and physical examination, while the case definition differed sometimes. Additionally, to determine the primary dysmenorrhea definition, three studies used a pain scale to assess the severity of dysmenorrhea. In more than half of the studies, women were recruited from gynecological clinics, which could indicate that the participants were women with a severe form of primary dysmenorrhea. The use of different laboratory analysis methods for markers in the various biologic samples (plasma, serum, neutrophils) did not allow us to perform a meta-analysis and a direct comparison of the results. Moreover, there was noticeable heterogeneity among participants from different studies, consisting of wide mean age, BMI, and sample size. Therefore, taking into account all these determinants, the results must be interpreted carefully.

The limitations of our study include a relatively small number of studies included in the review, a limited number of markers examined in relation to primary dysmenorrhea, especially regarding the antioxidant status and inflammation, and the fact that the majority of studies were performed in one country, Turkey.

### 4.3. Implications of Findings

Our findings have important public health and clinical implications, as primary dysmenorrhea affects up to 90% of menstruating women depending on the country, age, etc. [6]. The current findings may provide support for understanding primary dysmenorrhea, but no routine measurements of oxidative stress, antioxidant status, and inflammation markers can be recommended at the moment for the clinical diagnosis of primary dysmenorrhea. This review highlights a limited number of studies, especially on the antioxidant status, and a lack of studies with inflammation markers in relation to primary dysmenorrhea. Furthermore, there is a lack of studies in relation to primary dysmenorrhea in regions of the world other than Eurasia. Any further studies should aim to analyze in more detail associations between primary dysmenorrhea and a wider range of markers, especially inflammatory markers. Additionally, researchers should use a clear case definition, larger study populations, describe in more detail when the blood samples were taken (on which day of the menstrual cycle), and adjust the obtained results for potential confounders, such as age, BMI, or physical activity.

## 5. Conclusions

In conclusion, this systematic review indicates a higher level of oxidative stress in women with primary dysmenorrhea and limited evidence indicates a potential, lower antioxidant status in these women. These findings are of public health importance because of the high prevalence of primary dysmenorrhea and its extensive consequences. Whether oxidative stress, antioxidant status, and inflammation may participate in the pathophysiology of this disorder warrants further study.

## Figures and Tables

**Table 1:** Characteristics of the included studies.

Authors	Country	Cases, n	Case Definition	Controls, n	Participants Characteristics	Age Cases (Years; Mean ± SD)	Age Controls (Years; Mean ± SD)	Biomarkers of Interest	Adjustment
Akdemir et al. [34]	Turkey	33	Fluctuating, spasmodic menstrual cramps, sometimes referred to as “labor-like” pains that begin only a few hours before or with the onset of menstrual flow; lasts 2–3 days; menstrual pain frequently accompanied by backache, nausea, vomiting, diarrhea *	29	Inclusion: >18 years old, regular menses for at least six months	25.9 ± 7.1	27.3 ± 6.1	ADMA, hsCRP	No
					Exclusion: irritable bowel syndrome, inflammatory bowel disease or fibromyalgia, known premature coronary artery disease, family history of premature coronary artery disease, diabetes mellitus, hypertension				
Aksoy et al. [35]	Turkey	28	VAS, the pain was within two or three years after menarche, periodical and began a few hours before the menstruation and continued for the first 3 days of the cycle	26	Inclusion: 18–30 years old, regular menstrual cycles (25–30 days), nulliparous, non-smoking, no alcohol consumption, normal ultrasound examination of uterus and adnexa	26.1 ± 3.97	27.4 ± 3.03	MDA, NO, HO1	No
					Exclusion: chronic inflammatory, circulatory or surgical diseases of the abdomen–pelvis, cardiac or pulmonary diseases, endocrine and metabolic diseases, pelvic pathology, BMI ≥ 30 kg/m^2^, using any analgesic within 24 h prior to the study				
Dikensoy et al. [36]	Turkey	30	OLDCART mnemonic as abdominal pain or lower back pain during menstrual bleeding, and as two or more days of primary dysmenorrhea during menstrual bleeding; history of primary dysmenorrhea dating back to within one year of menarche, five-point scale ranging from 0–4	30	Inclusion: 20–34 years old, healthy women, regular menses for at least six previous cycles, nulliparous, non-smoking, non-drinking, acceptable method of barrier contraception	21 ± 0.2	20 ± 1.2	MDA, NO, AM	No
					Exclusion: intrauterine contraceptive device or an oral contraceptive, organic disorder which may cause dysmenorrhea				
Kaplan et al. [37]	Turkey	6	The initial onset of primary dysmenorrhea is usually six to 12 months after menarche, with the onset of ovulatory cycles. Lower abdominal or pelvic pain often occurs for eight to 72 h and is usually associated with the onset of menstrual flow. Back and thigh pain, headache, diarrhea, nausea, and vomiting may also be present (Proctor and Farquhar, 2006)	6	Inclusion: regular menstrual cycle *	23.3 ± 2.3	22.6 ± 2.4	LP, TAS, GSH, GSH-Px	No
					Exclusion: inflammatory disease, fibromyalgia, premature coronary artery disease, diabetes, mellitus, or hypertension, undergoing hormone replacement therapy, vitamin or mineral supplements for 6 months, smoking, drinking alcohol				
Orimadegun et al. [38]	Nigeria	45	Self-reported experience of lower abdominal/pelvic pain within 6–12 h of onset of menses and lasted 8–72 h in the 6-month period preceding the study	45	Inclusion: Regular menses for at least six previous cycles. Additional for controls: had never experienced lower abdominal/pelvic pain within 6–12 h of menstruation associated with onset of menses and lasted 8–72 h as well as history of illness or clinic consultation in 2 weeks preceding contact with the investigator	22.5 ± 3.3	21.8 ± 2.8	MDA, 3-NT, PrCarb, α-tocopherol	No
					Exclusion: history of recent alcohol consumption, cigarettes smoking, BMI > 25 kg/m^2^				
Turhan et al. [30]	Turkey	33	Onset of pain within 6–12 h after start of menstruation; lower abdominal or pelvic pain associated with onset of menses and lasting 8–72 h.; lower back pain during menses; medial or anterior thigh pain; menstrual pain with associated features such as headache, diarrhea, nausea and vomiting; 5-point pain scale ranging from 0 to 4	25	Inclusion: 21–32 years old, nulliparous, regular menses for at least 6 months, BMI < 23 kg/m^2^, acceptable method of barrier contraception	24.2 ± 3.1	25.0 ± 2.9	8-OhdG, 3-NT, SOD, MDA	No
					Exclusion: use of an intrauterine contraceptive device or an oral contraceptive, pelvic pathology, a history of alcohol and tobacco use; no analgesic within 24 h prior to the study				

3-NT, nitrotyrosine; 8-OhdG, deoxyguanosine; ADMA, asymmetric dimethylarginine, AM, adrenomedullin; BMI, body mass index; hs-CRP, high sensitivity C-reactive protein; GSH, glutathione; GSH-Px, glutathione peroxidase; HO1, heme oxygenase; Hsp-27, heat shock protein 27; LP, lipid peroxidation; MDA, malondialdehyde; NO, nitric oxide; OLDCART, onset, location, duration, characteristics, aggravating factors, relief, treatment; PrCarb, protein carbonyls; SD, standard deviation; SOD, superoxide dismutase; TAS, total antioxidant status; VAS, visual analog scale. * information obtained by direct contact with authors.

**Table 2:** Quality assessment of studies included to the systematic review.

Authors	Selection (Max. 4 Stars)	Comparability (Max. 2 Stars)	Exposure (Max. 3 Stars)	Total Points (Max. 9 Stars)	Quality Assessment ^a^
Akdemir et al. [34]	1	2	1	4	Medium
Aksoy et al. [35]	3	2	2	7	High
Dikensoy et al. [36]	2	2	2	6	Medium
Kaplan et al. [37]	2	1	1	4	Medium
Orimadegun et al. [38]	4	1	2	7	High
Turhan et al. [30]	2	2	2	6	Medium

**^a^** Quality assessment according to Newcastle–Ottawa Quality Assessment Scale [32]; 0–3 points were considered of low quality, from 4–6 points, studies were considered of medium quality, and from 7–9 points studies were considered of high quality.

**Table 3:** Oxidative stress and inflammation markers in women with dysmenorrhea and controls.

Biomarker	Authors	Unit	Biologic Sample	No. of Cases	No. of Controls	Mean ± SD, Cases	Mean ± SD, Controls	*p*-Value
LIPID PEROXIDATION								
MDA	Aksoy et al. [35]	μmol/mL	Serum	28	26	23.2 ± 3.46	20.9 ± 2.91	0.012
	Dikensoy et al. [36]	N/A	Plasma	30	30	2.75 ± 0.22	2.04 ± 0.14	<0.05
	Orimadegun et al. [38]	nmol/mL	Plasma	45	45	0.75 ± 0.19	0.45 ± 0.11	<0.001
	Turhan et al. [30]	nmol/mL	Plasma	33	25	1.32 ± 0.46	0.91 ± 0.26	<0.001
LP	Kaplan et al. [37]	μmol/g protein	Neutrophil	6	6	6.97 ± 0.51	5.63 ± 0.73	<0.01
PROTEIN and DNA DAMAGE								
3-NT	Orimadegun et al. [38]	ng/mL	Plasma	45	45	45.9 ± 37.1	21.3 ± 13.9	<0.001
	Turhan et al. [30]	nmol/mL	Plasma	33	25	81.4 (49.6–241) *	81.4 (53.6–117) *	0.489
PrCarb	Orimadegun et al. [38]	nmol/mg	Plasma	45	45	1.53 ± 0.27	1.50 ± 0.47	0.187
8-OHdG	Turhan et al. [30]	ng/mL	Serum	33	25	25.9 ± 3.89	27.8 ± 3.25	0.051
OTHER								
NO	Aksoy et al. [35]	nmol/mL	Serum	28	26	5.59 ± 2.32	3.96 ± 2.04	0.009
	Dikensoy et al. [36]	N/A	Plasma	30	30	43.6 ± 6.13	36.9 ± 3.24	<0.05
ADMA	Akdemir et al. [34]	μmol/L	Serum	33	29	0.31 (0.28–0.35) *	0.27 ± 0.09 *	0.025
Hs-CRP	Akdemir et al. [34]	mg/dL	Serum	33	29	0.1 (0.1–0.2) *	0.14 (0.1–0.15) *	0.225

3-NT, nitrotyrosine; 8-OhdG, deoxyguanosine; ADMA, asymmetric dimethylarginine; hs-CRP, high sensitivity C-reactive protein; LP, lipid peroxidation; MDA, malondialdehyde; N/A, not available; NO, nitric oxide; PrCarb, protein carbonyls; SD, standard deviation; * values are expressed as a median (interquartile range).

**Table 4:** Antioxidant status markers in women with dysmenorrhea and controls.

Biomarker	Authors	Unit	Biologic Sample	No. of Cases	No. of Controls	Mean ± SD, Cases	Mean ± SD, Controls	*p*-Value
TOTAL ANTIOXIDANT STATUS								
TAS	Kaplan et al. [37]	μmol H_2_O_2_ equiv/g protein	Neutrophil	6	6	3.77 ± 0.55	4.98 ± 1.19	<0.05
ENZYMATIC MARKERS								
SOD	Turhan et al. [30]	U/mL	Plasma	33	25	14.7 (10.6–23.6) *	14.7 (14.0–21.8) *	0.956
HO1	Aksoy et al. [35]	ng/mL	Serum	28	26	5.36 ± 1.57	3.94 ± 0.97	<0.001
GSH-Px	Kaplan et al. [37]	U/g protein	Neutrophil	6	6	4.07 ± 0.62	4.74 ± 0.99	<0.05
NON-ENZYMATIC MARKERS								
GSH	Kaplan et al. [37]	μmol/g protein	Neutrophil	6	6	1.78 ± 0.21	1.73 ± 0.09	>0.05
AM	Dikensoy et al. [36]	N/A	Plasma	30	30	33.8 ± 2.3	30.8 ± 2.5	<0.05
Vitamin E	Orimadegun et al. [38]	μmol/L	Plasma	45	45	7.51 ± 1.95	8.98 ± 1.95	<0.001

AM, adrenomedullin; GSH, glutathione; GSH-Px, glutathione peroxidase; HO1, heme oxygenase; N/A, not available; SD, standard deviation; SOD, superoxide dismutase; TAS, total antioxidant status * values are expressed as a median (interquartile range).

## Supplementary Materials

The following are available online at https://www.mdpi.com/2076-3921/9/10/994/s1, Table S1. Search term strategy, Table S2. Details of quality assessment of case-control studies according to The Newcastle-Ottawa Scale (NOS).
