# Supplementary material for: Primary Dysmenorrhea in Relation to Oxidative Stress and Antioxidant Status: A Systematic Review of Case-Control Studies
Source: Antioxidants (Basel). 2020 Oct 15;9(10):994. doi: 10.3390/antiox9100994 (PMC7602455; doi:10.3390/antiox9100994)
Supplement: Supplementary file 1 [file antioxidants-09-00994-s001.pdf]

SUPPLEMENTARY MATERIAL

Primary dysmenorrhea in relation to oxidative stress and antioxidant status: a systematic review of case-control studies

Maria Karolina Szmidt[*], Dominika Granda, Ewa Sicinska, Joanna Kaluza

***Corresponding author:** Maria Karolina Szmidt, Institute of Human Nutrition Sciences, Warsaw University of Life Sciences – SGGW, 159C Nowoursynowska Str., 02-776 Warsaw, Poland, E-mail: maria_szmidt@sggw.edu.pl, Tel: +48 22 59 37 119

Affiliations:

Institute of Human Nutrition Sciences, Warsaw University of Life Sciences–SGGW, 02-776 Warsaw, Poland

Table S1. Search term strategy

Search number	Search term	Pubmed – number of results	Scopus – number of results
#1	dysmenorrhea OR primary dysmenorrhea	6,926	12,608
#2	(((((((((((((((((((((inflammation) OR (dna damage)) OR (oxidative stress)) OR (antioxidant capacity)) OR (lipid peroxidation)) OR (catalase)) OR (glutathione peroxidase)) OR (glutathione reductase)) OR (isoprostane)) OR (nitric oxide)) OR (8-hydroxyguanosine)) OR (superoxide dismutase)) OR (malondialdehyde)) OR (hs-crp)) OR (nitrotyrosine)) OR (deoxyguanosine)) OR (interleukin-6)) OR (4-hydroxynonenal)) OR (protein carbonyl content)) OR (advanced glycation end products)) OR (advanced oxidation protein products)) OR (TNF-alpha)) OR (antioxidants)) OR (antioxidant status))	1,704,802	1,739,506
#3	#1 AND #2	317	618

Table S2. Details of quality assessment of case-control studies according to The Newcastle-Ottawa Scale (NOS)[32]

Authors	Is the case definition adequate?	Representativeness of the cases	Selection of Controls	Definition of Controls	SELECTION (max. 4 points)	Comparability for the most important factor - age	Comparability for any additional factor	COMPARABILITY (max. 2 points)	Ascertainment of exposure	Same method of ascertainment for cases and controls	Non-response rate	EXPOSURE (max. 3 points)	TOTAL POINTS (max. 9 points)
**Akdemir et al.***[34]	0	0	0	1	**1**	1	1	**2**	0	1	0	**1**	**4**
**Aksoy et al.** [35]	1	1	0	1	**3**	1	1	**2**	0	1	1	**2**	**7**
**Dikensoy et al.** [36]	1	0	0	1	**2**	1	1	**2**	1	1	0	**2**	**6**
**Kaplan et al.** [37]	1	0	0	1	**2**	1	0	**1**	0	1	0	**1**	**4**
**Orimadegun et al.**. [38]	1	1	1	1	**4**	1	0	**1**	1	1	0	**2**	**7**
**Turhan et al.** [30]	1	0	0	1	**2**	1	1	**2**	1	1	0	**2**	**6**

*Quality assessment was done based on published version of article. The information about case definition was later obtained via mail directly from authors.
